# Socioeconomic disparities and concentration of the spread of the COVID-19 pandemic in the province of Quebec, Canada

**DOI:** 10.1186/s12889-023-15983-3

**Published:** 2023-06-06

**Authors:** Gabrielle Lefebvre, Slim Haddad, Dominique Moncion-Groulx, Mélanie Saint-Onge, André Dontigny

**Affiliations:** 1 Direction de santé publique du CIUSSS-CN, Quebec City, QC Canada; 2 Centre de Recherche en Santé Durable VITAM, Quebec City, QC Canada; 3 Department of Geography, Université Laval, Quebec City, QC Canada

**Keywords:** COVID-19, Spatial inequalities in health, Social inequalities in health, Population density, Socioeconomic factors

## Abstract

**Background:**

Recent studies suggest that the risk of SARS-CoV-2 infection may be greater in more densely populated areas and in cities with a higher proportion of persons who are poor, immigrant, or essential workers. This study examines spatial inequalities in SARS-CoV-2 exposure in a health region of the province of Quebec in Canada.

**Methods:**

The study was conducted on the 1206 Canadian census dissemination areas in the Capitale-Nationale region of the province of Quebec. The observation period was 21 months (March 2020 to November 2021). The number of cases reported daily in each dissemination area was identified from available administrative databases. The magnitude of inequalities was estimated using Gini and Foster-Greer-Thorbecke (FGT) indices. The association between transmission and socioeconomic deprivation was identified based on the concentration of transmission in socially disadvantaged areas and on nonparametric regressions relating the cumulative incidence rate by area to ecological indicators of spatial disadvantage. Quantification of the association between median family income and degree of exposure of dissemination areas was supplemented by an ordered probit multiple regression model.

**Results:**

Spatial disparities were elevated (Gini = 0.265; 95% CI [0.251, 0.279]). The spread was more limited in the less densely populated areas of the Quebec City agglomeration and outlying municipalities. The mean cumulative incidence in the subsample made up of the areas most exposed to the pandemic was 0.093. The spread of the epidemic was concentrated in the most disadvantaged areas, especially in the densely populated areas. Socioeconomic inequality appeared early and increased with each successive pandemic wave. The models showed that areas with economically disadvantaged populations were three times more likely to be among the areas at highest risk for COVID-19 (RR = 3.55; 95% CI [2.02, 5.08]). In contrast, areas with a higher income population (fifth quintile) were two times less likely to be among the most exposed areas (RR = 0.52; 95% CI [0.32, 0.72]).

**Conclusion:**

As with the H1N1 pandemics of 1918 and 2009, the SARS-CoV-2 pandemic revealed social vulnerabilities. Further research is needed to explore the various manifestations of social inequality in relation to the pandemic.

**Supplementary Information:**

The online version contains supplementary material available at 10.1186/s12889-023-15983-3.

## Introduction

The coronavirus pandemic will leave its mark on contemporary history for its scale and the magnitude of its consequences. However, while all populations are affected, certain social groups are, because of their fragility, their work, or the precarious nature of their living conditions, at greater risk than others of becoming infected, developing severe forms of the disease, or dying from the 2019 coronavirus (COVID-19). The coronavirus pandemic appears to reflect the prevailing social hierarchies in health: the risk of severe acute respiratory syndrome coronavirus 2 (SARS-CoV-2) infection increases with poverty, lower educational attainment, unfavourable housing conditions, overcrowding, or speaking a language other than the country’s official language [1]. People with low incomes, living below the poverty line, or with precarious jobs were disproportionately affected by COVID-19 [2–4]. These social groups appear doubly disadvantaged. They are more frequently afflicted with co-morbidities—obesity, diabetes, cardiovascular diseases—that are themselves associated with COVID-19 [5–7] and more exposed, by their occupations, to the risk of contamination. Their jobs tend to be concentrated in sectors—logistics, distribution, manufacturing, transportation—that are not conducive to observing distancing measures or to teleworking [8]. These authors cite a British report, which notes that these populations, “are more likely to be working in the low paying jobs which are keeping the country going in supermarkets, as cleaners, delivery drivers and home care workers, and a significant proportion of these low paid workers will be women.” A systematic review concluded that exposure to SARS-CoV-2 was higher among people unable to work at home or stock up on food in their homes and among homeless populations, and that the economic impacts of the health crisis have disproportionately affected women and vulnerable populations [3]. Among these vulnerable populations are migrant populations and racial minorities [3]; in a Finnish study, the "income gradient in incidence" was mainly observed among migrant populations [9].

The unequal spatial distribution of populations naturally lays the foundation for spatial inequalities. According to Alidadi and Sharifi, “the driving forces [of COVID-19 spread] can be divided into seven main categories: density, land use, transportation and mobility, housing conditions, demographic factors, socio-economic factors, and health-related factors” [2]. Several studies have already reported on overexposure to the coronavirus in cities or areas with a high concentration of disadvantaged populations. A pan-Canadian study of the first two waves of the pandemic showed an overrepresentation of COVID-19 cases in cities with lower incomes and education levels and in those with a higher proportion of visible minorities, recent immigrants, high-density housing, and essential workers [10]. In Israel, during the first year of the pandemic, the risk of coronavirus infection was two times lower in cities with well-off populations (RR = 0.45; 95% CI [0.33, 0.62]) [11]. In England, the 79 territories reporting more daily cases of SARS-CoV-2 were notable for their higher levels of social and material deprivation, high population density, and higher avoidable mortality [12]. In the United States, several studies have demonstrated a significant association between mortality and the incidence of COVID-19 and the CDC’s Social Vulnerability Index (SVI) [13–21]. Each additional percentage point of farmworkers in a county was associated with 5.79 more deaths (5.51 directly, 0.28 via indirect ‘spillover’ to the next county, *p* <0.001), while each additional percentage point of individuals living in poverty was associated with 4.41 additional deaths (4.20 directly, 0.22 indirect, *p* <0.001) [22]. These results are consistent with findings from different parts of the world at different periods of the pandemic [2–4, 8, 23–26].

Population density is another key factor in COVID-19 transmission. Studies in densely populated countries, including China, Bangladesh, and the United States, have shown that population density has played an important role in virus transmission in urban areas [27–29]. It was a major determinant in nearly two-thirds of the 166 studies reviewed by Alidadi and Sharifi, as well as in the systematic review of spatial and spatiotemporal analyses of COVID-19 epidemiology carried out by Nazia et al. [2, 4]. According to the authors of this review of 154 articles, the association between transmission and population density has been found with great consistency all over the world, regardless of the methods and spatial analysis models used in these studies [4]. The literature suggests that population density plays a more pronounced determining role in more urbanized neighborhoods, cities, or districts [15, 24] and in settings where transmission and outbreaks are facilitated by the impossibility of isolation or by overcrowding [30].

Studies carried out to date thus provide a convincing overall picture of territorial inequalities and of the overexposure of disadvantaged populations and densely populated habitats to the disease and its most severe consequences. It should be noted, however, that the magnitude and potential strength of these associations vary substantially across studies, depending on the contexts, the selected social deprivation indicators, and of course, the levels of aggregation of spatial data (e.g., country, state, region, county) [31]. A comparison of ecological studies on the association between the CDC’s Social Vulnerability Index and COVID epidemiology in the United States provides an interesting illustration. The nine studies we consulted provided rather contrasting results, even though they were carried out in the same country, using the same unit of analysis (the county), and the same vulnerability indicator. One study in particular reported crossover situations; depending on the time of measurement, inequality appeared to be sometimes to the detriment and sometimes to the advantage of the most socially disadvantaged counties [15]. Similar findings were reported in a recent longitudinal study in Germany [32] and in a scoping review [23]: more than half of the 93 analyses that combined different socioeconomic indicators with COVID-19 outcomes found crossover dynamics in socioeconomic inequalities.

The heterogeneity of the estimates calls for spatiotemporal explorations based on sufficiently detailed data aggregation levels in order to identify local dynamics effectively and avoid the dilution effects inherent in heterogeneous spatial aggregates [31]. Unfortunately, there are not yet enough studies carried out at these levels [31, 33]. Secondly, longitudinal research designs are required with observation windows wide enough to identify the temporal dynamics of social inequalities over the entire pandemic and to verify their persistence, amplification, or attenuation, or even alternating patterns. However, the vast majority of studies have been cross-sectional, each carried out at a particular time during the pandemic, thereby contributing to the inconsistent results [15]. With a few exceptions [15, 31, 32], the longitudinal studies we were able to consult were based on narrow observation windows, including, at most, only a few waves or pivotal moments of the pandemic. Thus, of the 46 studies reported by Beese et al. in their scoping review, only one had an observation window of more than one year, and the observations lasted on average less than six months (mean = 23.4 weeks, with a period range of 3.9–59.9 weeks) [23].

This longitudinal study focused on spatial disparities in exposure to SARS-CoV-2 in the Capitale-Nationale health region of the province of Quebec in Canada. The first objective of the analysis was to estimate the extent of spatial inequalities and their evolution since the first case was reported in the region in March 2020. This included identifying areas of excess vulnerability characterized by particularly high levels of contagion. In order to identify the spatiotemporal dynamics as precisely as possible and to have consistent estimators, the approach was based on a very wide observation window of 90 weeks and, as spatial aggregates, micro-territories made up of the Canadian census dissemination areas. The second objective was to study the socioeconomic roots of spatial inequalities and the geographic concentrations of the pandemic’s spread. Overexposure to the coronavirus was first examined in relation to population density and several indicators reflecting the degree of socioeconomic deprivation of these populations. Further explorations were then conducted to better qualify the relationship between the levels of cumulative incidence and socioeconomic deprivation, after controlling for population density and other possible influential factors. We finally questioned the evolution of the economic gradient in health, wondering whether it declined, was maintained or were exacerbated overtime.

## Methods

### Context

The study covered the period from March 14, 2020 (first case identified in the region) to November 6, 2021, which encompassed the first four locally defined pandemic waves. The variable of interest was the cumulative incidence of disease (number of reported cases per capita) in each micro-territory defined by a Canadian census dissemination area (DA). Statistics Canada defines a DA as “the smallest standardized geographic area for which all census data are released” [34]. The population of a DA is usually between 400 and 700. At the last census, there were 1,206 DAs in the region, spread over six regional municipalities. The population is estimated to be over 750,000 in 2022, with four-fifths of the population living in the Quebec City agglomeration [35].

### Measurements

Data on daily reported cases in the region were drawn from administrative databases available from the regional public health department. Demographic and socioeconomic data aggregated by area were provided by the 2016 Canadian census and updates available on the Statistics Canada website. To avoid estimates being biased by the spatial concentration of settings in which certain clienteles or housing conditions excessively facilitate transmission, cases reported in penitentiaries and long-term care hospitals housing frail elderly people were excluded from the analyses. The population numbers and density of the DAs and three ecological indicators reflecting the socioeconomic level of those areas were derived from the census: the median income of economic families, the material deprivation index, and the social deprivation index [36]. The deprivation indices have been developed and used by the government and researchers in Quebec to analyze social inequalities in health [37]. The first includes three indicators—employment, education, and income—reflecting “the deprivation of goods and amenities of daily life of people residing in a territory and resulting in a lack of material resources.” The second also includes three indicators that are presumed to reflect the fragility of the social network—proportion of people living alone, single parents or separated, divorced or widowed). Both indices are derived using principal component analysis [36].

### Estimating spatial inequalities in health

**Fig. 1:**
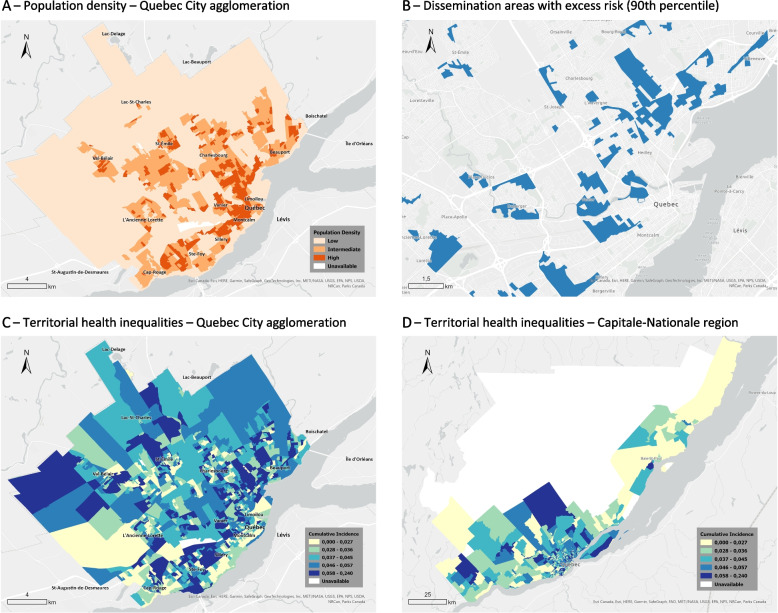
Spatial distribution of population and SARS-CoV-2 transmission. Quebec City agglomeration and Capitale-Nationale region

The inequality between territories was identified by examining the distribution in the whole sample and then in a subsample made up of the areas most exposed to the pandemic (last decile of the distribution). The measurement of inequality between areas was provided by the Gini index, a standardized measure that ranges within the interval [0,1]. A zero value for the index would reflect perfect equality of cumulative incidence among DAs. As inequality increases, so does the value of the index. Because of the heterogeneity of demographics and housing, the DAs were divided into four strata according to their location and population density. The first three are located in the Quebec City regional county municipality (Fig. 1A). The fourth is composed of areas located in the outlying municipalities.

Foster-Greer-Thorbecke (FGT) indices [38], initially developed for poverty analysis and then extended to the analysis of health inequality [39, 40], were used to estimate the extent of the spread and to explore the levels of excess risk encountered in the areas most exposed to the epidemic. In this case there were 119 areas, which we will refer to as areas at excess risk, where the cumulative incidence was greater than 0.069, i.e., the incidence at the 90^th^ percentile of the distribution.^1^ FGT_0_ equalled the proportion of units with excess risk. FGT_1_ reflected the mean excess risk of the group consisting of the 119 areas at excess risk. It was equal to the mean difference in these areas between the cumulative incidence and the chosen threshold value. The index was expressed in relative values; a value of 10% meant, for example, that the cumulative incidence of the areas at excess risk was, on average, 10% higher than the incidence observed at the 90^th^ percentile of the distribution. FGT_2_ was an estimator that also focused on the subsample of areas at excess risk. It reflected the inequality between these vulnerable areas.

### Analysis of social health inequalities

The association between transmission and socioeconomic deprivation in the DAs was explored through a series of analyses. Preliminary examination of the data showed that these associations varied significantly within the group consisting of outlying municipalities (Supplementary Fig. 1). The limited number of DAs included in each of these municipalities also constrained our ability to conduct in-depth quantitative analyses. Consequently, these analyses were conducted on a subsample consisting of the 946 DAs in the Quebec City agglomeration.

Non-linear regressions were used first to explore the relationship between the cumulative incidence rate by area and ecological measures of spatial deprivation. The FGT indices were then compared across DAs showing different levels of socioeconomic deprivation. This was a five-modality categorical variable, constructed by ranking DAs by family income and then grouping them into quintiles. The first quintile was made up of areas with the lowest income and the last quintile, of those with the highest income. Concentration coefficients were estimated to assess the concentration of transmission in the most disadvantaged areas. Like the Gini index, the concentration coefficient has a zero value in the absence of social inequality in health. Its value increases as the association between levels of cumulative incidence and socioeconomic deprivation increases. A negative value reflects a concentration of transmission in the lowest income strata. The quantification of the association between median family income and the degree of exposure of the DAs was supplemented by an ordered probit multiple regression model. The dependent variable was constructed based on the level of cumulative incidence. This was a five-modality ordinal variable, the DAs being divided into five groups of equivalent size based on the value of their cumulative incidence. The first modality included the areas of the first quintile of the distribution (cumulative incidence less than 0.027). The second modality included the areas of the second quintile, and so on. The level of economic deprivation of the area presented in the previous paragraph (distribution quintile) was the independent variable of interest. The association between median family income and the level of exposure of the DAs was examined after controlling for the influence of the possible confounding factors of population density and size, municipality of residence, and proportion of immigrants. All analyses were performed using Stata 17 software [41].

## Results

### Spatial health inequalities

During the observation period, nearly all of the 1,206 dissemination areas in the region reported cases. The cumulative incidence per area averaged 4.4 reported cases per 100 residents (median = 4.1). Spread was more limited in municipalities outside the Quebec City metropolitan area and, within the metropolitan area, in less densely populated neighbourhoods (Table 1, Fig. 1, and Supplementary table 1). The cumulative incidence of the area at the 90^th^ percentile of the distribution was three times higher than that of the 10^th^ percentile (90/10 ratio = 3.37). The Gini index for all areas was equal to 0.265 (95% CI [0.251, 0.279]). The index varied little between strata, as did the 90/10 ratio. In other words, the spread differed significantly between territories, but the inequality between areas of equal population density was relatively constant. The second part of Table 1 focuses on the 119 areas at excess risk. The cumulative incidence was, on average, 0.093 (median = 0.083), i.e., a mean deviation (FGT_1_) from the threshold value of 35% (95% CI [26.0%, 43.5%]). These areas were located mainly, but not exclusively, in the most densely populated areas of downtown Quebec City (Fig. 1B). This group of areas was heterogeneous (Gini = 0.15; FGT_2_ = 33%), with cumulative incidence ranging from 0.069 to 0.24.

**Table 1:** Cumulative incidence^a^ and inequality indicators by location and population density of dissemination areas. Capitale-Nationale health region. Reported cases as of November 11, 2021

	All areas	Quebec City agglomeration population density			Other regional municipalities
		lower	intermediate	higher	
All areas with reported cases^a^					
Number	1188	171	381	394	242
Cumulative incidence^a^					
Mean	0.044	0.046	0.043	0.049	0.036
Median	0.041	0.042	0.041	0.045	0.034
Inequality					
10^th^ percentile	0.020	0.024	0.021	0.023	0.016
90^th^ percentile	0.069	0.070	0.067	0.078	0.056
Ratio 90/10	3.37	2.88	3.14	3.36	3.49
Gini	0.265	0.250	0.246	0.263	0.272
	[.251 – .279]	[ .211 – .288]	[ .226 – .266]	[.237 – .290]	[.242 –.301]
Dissemination areas with excess risk^b^					
Number	119	19	34	57	9
As a proportion of areas in the zone (FGT_0_)	10%	11%	9%	14.5%	3.7%
Cumulative incidence					
Mean	0.093	^c^	0.088	0.095	^c^
Median	0.083	^c^	0.079	0.084	^c^
Standard deviation at 90^th^ percentile (FGT_1_^c^)	35.3%				
	[27.0% – 43.5%]				
Inequality					
Gini	0.151				
	[.120 – .182]				
FGT_2_^c^	32.5%				
	[17.5% – 47.6%]				

^a^ All reported cases excluding persons residing in long-term care hospitals and correctional facilities

^b^Areas with cumulative incidence above the 90^th^ percentile of the distribution (*p* = 0.067)

^c^Insufficient numbers to calculate precise estimators

### Social and spatial health inequalities

Table 2 provides a summary picture of the socioeconomic characteristics of the DAs by cumulative incidence. The areas were divided into four groups according to whether they fell between the 1^st^ and 10^th^ percentile of the distribution, the 10^th^ percentile and the median, the median and the 90^th^ percentile, or above the 90^th^ percentile. The comparison revealed the significant concentration of spread in the most disadvantaged territories. The most exposed territories were those in which the indices of social and material deprivation were highest. Median income was lower in the most exposed areas and vice versa. The proportion of immigrants and the population density in the most affected DAs (90^th^ percentile) exceeded that of the least exposed areas by almost 50%, and the median income of economic families was 20% lower.

**Table 2:** Socioeconomic characteristics of the dissemination areas by cumulative incidence

	All areas	Dissemination areas grouped by cumulative incidence				
Median		1st-10^th^ percentile (1)	10th-50^th^ percentile (2)	50th-90^th^ percentile (3)	90th-100^th^ percentile (4)	Ratio (4)/(1)
Population density per km^2^	2 751	2 319	2 519	2 923	3 395	1.46
Proportion of immigrants in the population	0.041	0.032	0.038	0.045	0.046	1.44
Material deprivation index	0.018	-0.016	-0.023	-0.018	-0.002	
Social deprivation index	0.000	-0.009	-0.006	0.003	0.010	
Median income of economic families	82 944	86 400	86 336	81 664	69 846	0.81

**Fig. 2:**
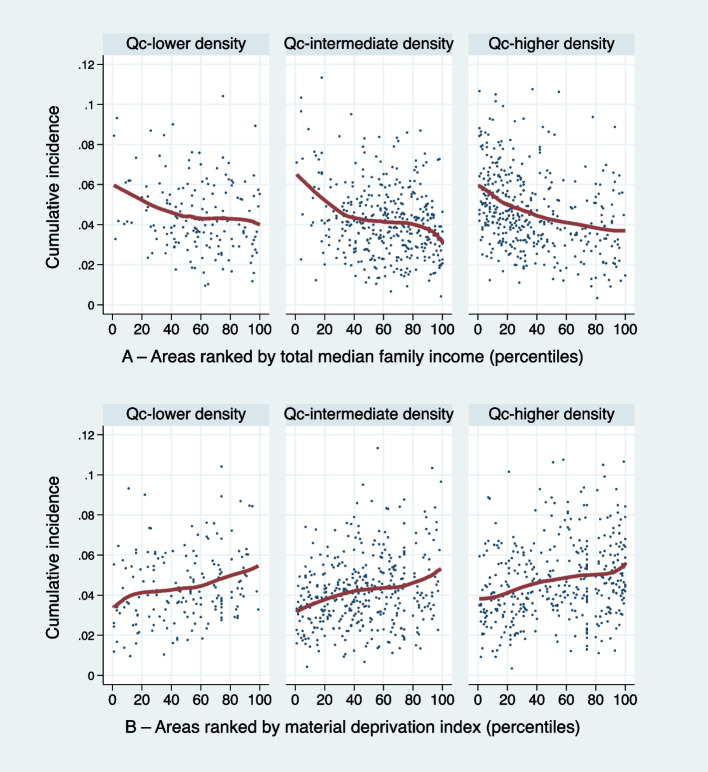
Cumulative incidence and material and economic deprivation by population density. Subsample of dissemination areas in the Quebec City agglomeration (*N* = 946)

The curves presented in Fig. 2 present the results of non-parametric regressions on the relationship between cumulative incidence and ecological indicators of deprivation. The analysis was repeated in each of the strata defined by the area locations and population density. In the three strata of the Quebec City agglomeration, the economic gradient in health persisted after stratification. Exposure to the pandemic was associated with median household income and material deprivation in the Quebec City agglomeration. In contrast, the relationship between spread and social deprivation was less tangible (Supplementary Fig. 2).

In the following sections, median household income will be used as the reference indicator for calculating inequality indices. The concentration coefficients in the three zones were negative. They were -0.03 (95% CI [0.07, 0.00]) in the least densely populated areas, 0.07 (95% CI [0.10, 0.4]) in the intermediate stratum, and -0.09 (95% CI [0.12, 0.07]) in the most densely populated areas. These values thus indicated a concentration of the epidemic’s spread in economically disadvantaged areas, especially in densely populated areas.

**Fig. 3:**
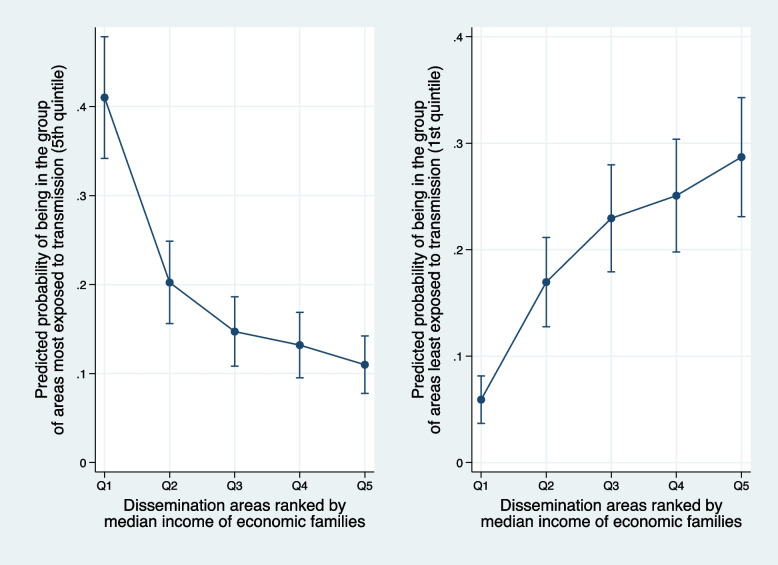
Probability of being in the most exposed or least exposed group of areas by level of economic deprivation of the area. Subsample of dissemination areas in the Quebec City agglomeration (*N* = 946)

Statistical modeling supplemented this picture of social inequalities in health. Based on the model, the probability that a more socioeconomically disadvantaged area (first income quintile) was in the group least exposed, or most exposed, to the epidemic was estimated. The process was then replicated for each income group. The results are shown in Fig. 3 (see Supplementary table 2). An area with an economically disadvantaged population was three times more likely to be among the areas most exposed to the pandemic (RR = 3.55; 95% CI [2.02, 5.08]), whereas an area with a higher income population was two times less likely to be among the areas most exposed to the pandemic (RR = 0.52; 95% CI [0.32, 0.72]). Conversely, an area with a low-income population had less than a 10% likelihood of being in the most spared quintile (RR = 0.09; 95% CI [0.07, 0.12]). An area with the highest median income population had a less than 26% likelihood of being in the most spared quintile (RR = 0.26; 95% CI [0.21, 0.31]).

### Evolution of the economic gradient in health

**Fig. 4:**
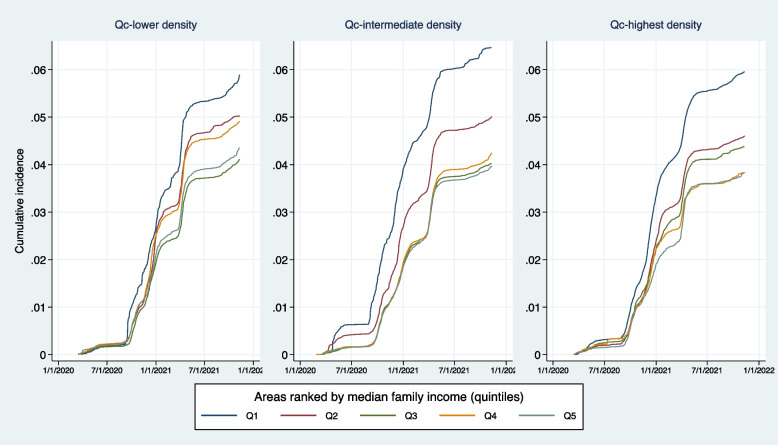
Changes in cumulative incidence by median income of families in dissemination areas and population density. Subsample of areas in the Quebec City agglomeration

Figure 4 suggests that socioeconomic inequality in health appeared from the beginning of the pandemic. It persisted and tended to increase thereafter, as the different waves were experienced. Over time, the areas with the lowest family incomes were demarcated from the other groups of areas, especially in the two strata that included the most densely populated areas.

### Economic deprivation and excess risk

Of the 119 areas at excess risk, 44% (*N* = 51) belonged to the group made up of the most socioeconomically disadvantaged territories (first income quintile), while only 10% (*N* = 12) belonged to the group consisting of the areas with the highest income (Table 3). In other words, an area with lower income was four times more likely to be in the excess risk group than an area with the highest family income. Among the areas with the lowest family income, more than one in five were in excess risk, even though they constituted only 10% of the areas (Table 3; FGT_0_ = 21.6%). Only one in 20 of the highest-income areas had excess risk.

**Table 3:** Socioeconomic inequalities (characteristics of transmission) among dissemination areas with excess risk^a^. Subsample of dissemination areas in the Quebec City agglomeration (*N* = 946)

	Most disadvantaged areas	Other areas	Most advantaged areas	Ratio (1) / (3)
	Q1 (*n* = 50) (1)	Q234 (*n* = 48) (2)	Q5 (*n* = 10) (3)	
FGT_0_	21.6%	7.5%	5.1%	4.21
	[16.3% – 26.8%]	[5.5% – 9.4%]	[2.3% – 8.0%]	
FGT_1_^a^	38.5%	30.1%	11.3%	3.40
	[24.0% – 52.9%]	[20.1% – 40.0%]	[3.9% – 18.8%]	
	40.7%	20.9%	2.6%	15.99
FGT_2_^a^	[10.4% – 71.1%]	[8.4% – 33.3%]	[0.2% – 4.9%]	

^a^Baseline: cumulative incidence at the 90^th^ percentile of the distribution

The mean excess risk in the economically disadvantaged areas was 38.1%, a value four times higher than that observed in the 12 areas with the highest family income (FGT_1_ = 9.9%). The cumulative incidence in the most economically advantaged areas was only slightly above the cut-off value (0.076 rather than 0.069). In other words, excess risk was modest in the more advantaged areas and greater in those with the lowest family income. The standard deviations and FGT_2_ indices also showed that disparities were more pronounced within the same group of economically more disadvantaged areas: FGT_2_ reached 40%, whereas at the opposite end of the spectrum, the group of areas at excess risk containing the population in the highest income bracket was extremely homogeneous (FGT_2_ = 2.1%).

## Discussion

This ecological study of social inequalities in the spread of the epidemic is based on territorial aggregates—dissemination areas—corresponding to the smallest standardized geographic area of the Canadian census whose data are available to researchers [34]. A dissemination area comprises 400 to 700 people residing in one or more neighboring blocks and therefore constitutes a relatively homogeneous geographical entity. The study is a priori less exposed to the dilution effects and imprecise estimators to which analyses conducted at the county, neighborhood, or regional level are exposed. Another advantage of this study is the long observation period chosen, covering 90 weeks from the start of the health crisis, which made it possible to track the evolution of the pattern of socioeconomic inequalities and to report on the cumulative incidence of COVID-19 cases from the first wave until the arrival of the Omicron strain, which caused a phenomenal jump in COVID-19 cases and made it virtually impossible to count new cases.

This study showed that transmission differed significantly across micro-territories. The Gini index exceeded 0.25, and the cumulative incidence of the area at the 90^th^ percentile of the distribution was more than three times higher than that at the 10^th^ percentile (0.093 vs. 0.044). The level of transmission tended to change with habitat density. The analyses also confirmed other studies and previous systematic reviews showing a strong relationship between the spread of the pandemic and urban population concentrations [2, 9, 24, 26–29, 42]. More densely populated urban areas were more frequently found to be among the areas most affected by the pandemic than less populated urban areas (14.5% vs. 10.5) or rural areas (3.7%). The combined effect of concentrated housing and the over-representation of disadvantaged social groups in urban areas naturally favoured transmission. However, the relationship between urban areas and transmission must also be considered in relation to the particular features of the local settings and, in particular, the availability and accessibility of public services (transport, social services, and especially local health care services). Excess mortality due to COVID-19 has been reported in rural counties in the United States. Mortality was more strongly associated with social determinants of health in rural counties than in urban counties, possibly due to excess mortality among farm workers. Other studies have reported excess transmission of COVID-19 in rural areas underserved by health services [8] or where the health care system was overburdened during certain critical phases of the pandemic [3].

Several lessons can be drawn from analyses of the relationship between the extent of transmission in micro-territories and economic disadvantage. First, as other studies have also shown, transmission was clearly concentrated in economically disadvantaged areas [3, 10, 28, 43]. It was higher in economically disadvantaged areas, even after controlling for population density and size, municipality of residence, and proportion of immigrants in the area. Areas with the lowest median family income (first quintile) were also over-represented among the most severely affected areas. One area in five was found to be in the subsample of areas at excess risk, even though this group included only 10% of the region’s territories, and their cumulative incidence exceeded that of the areas with the most economically advantaged families by an average of two percentage points (0.095 vs. 0.076).

Second, the overexposure of economically disadvantaged dissemination areas was evident from the start of the pandemic. The socioeconomic roots of transmission began to be apparent in the first months of the health crisis—before, for example, certain personal protection devices such as vaccination had been deployed. The inequality then gradually increased. Neither the containment measures imposed throughout the pandemic, nor improvements in knowledge and practices to control the epidemic, nor vaccination have been able to arrest or even to contain the rise in social inequality in health. A similar pattern has been reported in Spain [26]. In Germany, Rohleder et al. also observed an increase over time in the magnitude of the adjusted association between deprivation and COVID-19 incidence [32]. However, in their study, as in others [7, 15, 44], the association became positive only after the first wave; the most privileged areas were more affected in the first weeks of the pandemic. The authors hypothesize that the more affluent travelled more and may have been the driving force of transmission in the initial phases, and that subsequently the disadvantaged groups, who were more vulnerable, more exposed to infection risk through their employment, or less able to isolate themselves, took the lead. In the same vein, Beese et al. link these developments to government decisions affecting people’s mobility: the better-off, who were initially more mobile, were more able to respect the social distance measures and constraints imposed [23].

Third, our results confirm the presence of what Marmot and Allen refer to as a “gradient of disadvantage” [45]. In particular, nonparametric regressions suggest that the relationship between the cumulative incidence of dissemination areas and median family income has a descending monotonic pattern (Fig. 2A), while the relationship between the cumulative incidence of dissemination areas and the material deprivation index has an ascending monotonic pattern (Fig. 2B). These patterns are robust, replicating similarly across territories of high, medium, or low urban density. Figure 3 provides a second illustration of this social gradient; the risk of being among the micro-territories highly overexposed or, on the contrary, relatively spared by COVID-19, varies very directly with the income level of families in these territories. Marmot and Allen rightly point out that these inequalities are superimposed on pre-existing social inequalities in health, such that COVID-19 “exposes and amplifies inequalities in society” [45]. Interestingly, several authors have established that COVID-19 transmission is associated not only with household income, but also with income inequalities in the territories considered [8, 46, 47]. Our analyses conducted at the micro-territories level did not allow us to explore these aspects. However, two comparative analyses have shown that countries with the greatest income inequalities were the most affected by the pandemic, notably the United States, the United Kingdom, and Italy [48, 49]. According to his calculations, Davies estimated that “37 percent of the gap in cases [between Canada and the United States] and 28 percent of the gap in deaths between the two countries could be attributed to their difference in income inequality” [49]. Social protection policies reportedly played an important role in reducing the economic impact of the pandemic on low-wage workers, but their effectiveness has been limited in countries where these policies are less developed [48].

### Limitations

Insofar as it is based on territorial aggregates, the analysis of the geographical concentration of incident cases did not allow for a detailed account of inter-individual disparities related, for example, to age, gender, or social status. Second, in the analysis of reported cases performed here, it was not possible to assess potential disparities in access to care, occurrence of complications, or socioeconomic impacts of the health crisis, which would need to be explored by other research studies. However, inequality in exposure to the disease was a core component in the health inequalities related to this health crisis, and the evidence presented can be considered a first step in exploring the inequalities related to the epidemic. Third, the count of reported cases stopped in November 2021. This choice was dictated by the need to end the calculation of cumulative incidences before case counts became too inaccurate, as the Omicron strain became predominant and screening and reporting capacities were affected. A final limitation was the use of compositional socioeconomic data (median household income and aggregate indices of deprivation) to characterize DAs. These aggregates could mask possible disparities within the territories concerned. Under the circumstances, the choice to use dissemination areas, the finest micro-geographic units available, minimized the heterogeneity that could have been prevalent in the observation units.

## Conclusion

As with the H1N1 pandemics of 1918 and 2009, the SARS-CoV-2 pandemic appears to have revealed social vulnerabilities and the relationship between social hierarchy and health [18, 19, 45]. Far from being “the great equalizer” [50] affecting our populations indiscriminately, COVID-19 has been a powerful amplifier of the social gradient in health, disproportionately affecting the poor, migrants, minorities, and more generally those whose health or social condition contain multiple factors of vulnerability.

We believe these findings reinforce the constantly repeated call for integrating social determinants into public health policies and interventions in general, and into epidemic response plans in particular [51]. The magnitude of the disparities observed at the very small scale also highlights the need for intervention models anchored in local realities so as to provide, among other things, adequate access to tests, vaccines, treatments, protective devices, and other health inputs to populations at greater risk because of their socio-economic conditions [3, 23, 52]. Accordingly, epidemiological surveillance systems and local monitoring of territorial disparities in the transmission of infectious diseases should have the capacity to identify local disparities and spatial concentrations of transmission promptly and in as much detail as possible. This health information should then be coupled with social and economic ecological information to describe pockets of vulnerability and ensure local monitoring of social inequalities. It is only then, through a proactive anticipatory approach involving public services and community-based social service organizations [53], that coordinated actions, upstream of health crises, might be able to protect the populations concerned and reduce the snares of excess morbidity, exclusion, and poverty to which they are exposed.

The present study has lifted the veil on social inequalities in health related to the pandemic without, however, being able to capture all of its dimensions. Further research should carry forward the proposed approach, with a view to exploring the various manifestations of social inequality in relation to the SARS-CoV-2 pandemic, trying to understand its causes, and nurturing territory-based action that would target “excess” social and health vulnerabilities. We also need to better understand the shortcomings of our responses to the current pandemic: how we got here, and why our health systems, including supposedly progressive social protection systems, have not been able to stop the spiral of inequality, and of course, “how we should deal with such pandemics now and in the future” and anchor the fight against pandemics in an inclusive protection approach [54].

## Supplementary Information

**Additional file 1: Supplementary figure 1.** Cumulative incidence by median family income. Regional municipalities outside the Quebec City area. **Supplementary table 1.** Cumulative cases reported as of November 6, 2021, by regional county municipality*. Capitale-Nationale health region. **Supplementary figure 2.** Cumulative incidence and social deprivation by population density. Subsample of dissemination areas in the Quebec City agglomeration (*N* = 946). **Supplementary table 2.** Probability that an area will be in the group of areas most exposed or least exposed to the pandemic based on the area’s level of economic deprivation.

## Data Availability

Survey microdata are not publicly available**.** Aggregated statistics could be provided to researchers by the corresponding author.

## Footnotes

1 The exercise was also conducted on two other subsamples defined by the 80^th^ and 95^th^ percentiles of the distribution. This report presents only the results from the exercise performed on the 90^th^ percentile.
