# Supplementary material for: Socioeconomic disparities and concentration of the spread of the COVID-19 pandemic in the province of Quebec, Canada
Source: BMC Public Health. 2023 Jun 6;23:1096. doi: 10.1186/s12889-023-15983-3 (PMC10243257; doi:10.1186/s12889-023-15983-3)
Supplement: Supplementary file 1 — Additional file 1: Supplementary figure 1. Cumulative incidence by median family income. Regional municipalities outside the Quebec City area. Supplementary table 1. Cumulative cases reported as of November 6, 2021, by regional county municipality*. Capitale-Nationale health region. Supplementary figure 2. Cumulative incidence and social deprivation by population density. Subsample of dissemination areas in the Quebec City agglomeration (N = 946). Supplementary table 2. Probability that an area will be in the group of areas most exposed or least exposed to the pandemic based on the area’s level of economic deprivation. [file 12889_2023_15983_MOESM1_ESM.docx]

Additional file : Supplementary figure 1

Cumulative incidence by median family income. Regional municipalities outside the Quebec City area.

Additional file : Supplementary table 1

Cumulative cases reported as of November 6, 2021, by regional county municipality*.

Capitale-Nationale health region.

| Regional county municipality | Number of areas | Number of cases* |
| --- | --- | --- |
| Charlevoix-Est | 32 | 329 |
| Charlevoix | 22 | 376 |
| Ile d'Orléans | 11 | 357 |
| Côte de Beaupré | 41 | 1 216 |
| La Jacques-Cartier | 49 | 1 822 |
| Québec | 946 | 29 117 |
| Portneuf | 88 | 88 |
| Non-assigned | - | 55 |
| Total | 1 189 | 35 473 |

* All reported cases except those residing in long-term care hospitals and correctional facilities

Additional file : Supplementary figure 2

Cumulative incidence and social deprivation by population density.

Subsample of dissemination areas in the Quebec City agglomeration (N = 946).

Additional file : Supplementary table 2

Probability that an area will be in the group of areas most exposed or least exposed to the pandemic based on the area’s level of economic deprivation.

|  | Probability* that an area  will be in the group of areas: | | Probability ratios |
| --- | --- | --- | --- |
|  | most exposed** | least exposed*** |  |
| Family income stratum | |  |  |
| Most economically disadvantaged areas (Q1) | 0.33 | 0.09 | 3.55 |
|  | [.28; .39] | [.07; .12] | [2.02; 5.08] |
| Most economically advantaged areas (Q5) | .13 | .26 | .52 |
|  | [.10; .17] | [.21; .31] | [.32; .72] |

* Predicted probability of being in a given group of areas after adjusting for population density and size, municipality of residence, and proportion of immigrants in the area.

** First quintile of the distribution: cumulative incidence less than 0.027.

*** Fifth quintile of the distribution: cumulative incidence greater than or equal to 0.058.
